# Significance of common variants on human chromosome 8q24 in relation to the risk of prostate cancer in native Japanese men

**DOI:** 10.1186/1471-2156-10-37

**Published:** 2009-07-14

**Authors:** Miao Liu, Takayuki Kurosaki, Motofumi Suzuki, Yutaka Enomoto, Hiroaki Nishimatsu, Tomio Arai, Motoji Sawabe, Takayuki Hosoi, Yukio Homma, Tadaichi Kitamura

**Affiliations:** 1 Department of Urology, Graduate School of Medicine, The University of Tokyo, 7-3-1 Hongo, Bunkyo-ku, Tokyo 113-8655, Japan; 2 Department of Pathology, Tokyo Metropolitan Geriatric Hospital, 35-2 Sakae-cho, Itabashi-ku, Tokyo 173-0015, Japan; 3 Department of Advanced Medicine, National Center for Geriatrics and Gerontology, 36-3 Gengo, Morioka-cho, Ohbu, Aichi 474-8511, Japan; 4 Department of Urology, Asoka Hospital, 1-18-1 Sumiyoshi, Koutou-ku, Tokyo 135-0002, Japan

## Abstract

**Background:**

Common variants on human chromosome 8q24, rs1447295 (C/A) and rs6983267 (T/G), have been recently linked to the prevalence of prostate cancer in European and American populations. Here, we evaluated whether the single-nucleotide polymorphisms rs1447295 and rs6983267 were associated with the risk of sporadic prostate cancer as well as latent prostate cancer in a native Japanese population.

**Results:**

We analyzed genomic DNA samples from 391 sporadic prostate cancer patients, 323 controls who had died from causes unrelated to cancer and 112 Japanese men who were diagnosed as having latent prostate cancer based on autopsy results. The polymorphisms were determined by allelic discrimination using a fluorescent-based TaqMan assay. The A allele of rs1447295 was significantly associated with the risk of sporadic prostate cancer (*p *= 0.04; age-adjusted OR, 1.34), while the G allele of rs6983267 showed a trend towards being a high-risk allele (*p *= 0.06; age-adjusted OR, 1.27). No significant difference between these two polymorphisms and the risk of latent prostate cancer was observed in the present Japanese population.

**Conclusion:**

Known variants on human chromosome 8q24 may be risk factors for sporadic prostate cancer in native Japanese men.

## Background

The incidence of prostate cancer is widely known to be much lower in Asian men than in Western men [1]. Recently, genome-wide association studies have revealed a close relation between variants on human chromosome 8q24 and the risk of prostate cancer [2-13]. In 2006, Amundadottir et al. [2] first identified a region on chromosome 8q24 that was possibly linked to prostate cancer in Icelandic men. Subsequently, Freedman et al. [3] confirmed an association between rs1447295 and the risk of prostate cancer in another ethnic cohort study. In yet another study, the prevalence of the rs1447295 polymorphism associated with prostate cancer was investigated in a population of Indian-born Asian Indians who had emigrated from India and were living in the United States [14]. In 2007, Haiman et al. [8] and Yeager et al. [9] showed a strong association between rs6983267 and the risk of prostate cancer. Their investigations included African Americans, Latino Americans, European Americans, Japanese Americans, native Hawaiians, Australians, Swedish, Icelanders, and the British, but not native Japanese or other East Asian men. Thus, we felt that it was important to replicate the study in a population of native Japanese subjects to better understand the disparities in prostate cancer risk among different ethnicities.

The characteristics of germline genetic polymorphisms among patients with latent prostate cancer diagnosed at the time of autopsy (LPCa) remain unknown. Stamey et al. first proposed the pathologic entity of clinically diagnosed latent prostate cancers, which are defined as clinically insignificant cancers [15]. Strictly speaking, 'insignificant prostate cancer' is not the same as 'LPCa', and a comparative genetic analysis of clinically diagnosed sporadic prostate cancer (SPCa) and LPCa might be useful for a better understanding of the nature of this disease. From this viewpoint, we also included LPCa subjects in the present study to examine the genetic differences between SPCa and LPCa patients.

## Results

The characteristics of the control, SPCa, and LPCa groups are shown in Table 1. The age distributions of the control, SPCa, and LPCa groups were significantly different (*p *< 0.01). The T-stage distributions in the SPCa and LPCa groups were also significantly different (*p *< 0.01). The Gleason score distributions in the SPCa and LPCa groups were not significantly different (*p *= 0.49). Genotyping assays were successfully performed in all the subjects. Table 2 shows the distributions of the genotypes and alleles of the rs1447295 and rs6983267 polymorphisms. The genotype distributions for each SNP were consistent with Hardy-Weinberg equilibrium (HWE).

**Table 1:** Characteristics of control, SPCa, and LPCa

		Control (N = 323)	SPCa (N = 391)	LPCa (N = 112)	*p *value
Age					
	median	79	71	81	
	range	49 – 100	48 – 89	66 – 98	< 0.01
					
T-stage					
	T1 or T2	－	167	102	
	T3 or T4	－	224	5	< 0.01
	unknown	－	0	5	
					
Gleason score					
	≤7	－	241 (92)*	62	
	≥8	－	150 (34)*	45	0.49
	unknown	－	0	5	

*Numbers of patients with SPCa who were undergone radical prostatectomy are described in the parenthesis.

—, not applicable.

**Table 2:** Genotypic and allelic frequencies of rs1447295 and rs6983267 polymorphisms

Genotype	Control (%) (N = 323)	SPCa (%) (N = 391)	LPCa (%) (N = 112)
rs1447295			
CC	218 (67.5)	217 (55.5)	71 (63.4)
CA	89 (27.5)	149 (38.1)	34 (30.4)
AA	16 (5.0)	25 (6.4)	7 (6.2)

rs6983267			
TT	147 (45.5)	151 (38.6)	48 (42.9)
TG	151 (46.8)	181 (46.3)	53 (47.3)
GG	25 (7.7)	59 (15.1)	11 (9.8)

Allele	Control (%) (2N = 646)	SPCa (%) (2N = 782)	LPCa (%) (2N = 224)

rs1447295			
C	525 (81.3)	583 (74.6)	176 (78.6)
A	121 (18.7)	199 (25.4)	48 (21.4)
HWE			
chi-square	2.91	0.01	1.09
*p *value	0.09	0.93	0.30

rs6983267			
T	445 (68.9)	483 (61.8)	149 (66.5)
G	201 (31.1)	299 (38.2)	75 (33.5)
HWE			
chi-square	2.65	0.16	0.44
*p *value	0.10	0.69	0.51

HWE, Hardy-Weinberg equilibrium.

Compared with the major allele homozygous genotype as a reference, both the CA genotype of rs1447295 (*p *= 0.02; age-adjusted OR, 1.54; 95% CI, 1.08 – 2.21) and the GG genotype of rs6983267 (*p *= 7.0 × 10^-3^; age-adjusted OR, 2.21; 95% CI, 1.24 – 4.03) were significantly associated with the risk of SPCa. Moreover, the CA + AA genotypes of rs1447295 was also significantly associated with the risk of SPCa (*p *= 0.02; age-adjusted OR, 1.50; 95% CI, 1.07 – 2.11). In allele-wise analyses, the A allele of rs1447295 was significantly associated with the risk of prostate cancer (*p *= 0.04; age-adjusted OR, 1.34; 95% CI, 1.01 – 1.79), while the G allele of rs6983267 showed a tendency towards an increase in the risk of prostate cancer (P = 0.06; age-adjusted OR, 1.27; 95% CI, 0.99 – 1.62). One hundred and twelve patients among 1,179 autopsied men were diagnosed as having LPCa (9.5%). No significant differences were observed between the control and the LPCa patients (Table 3).

**Table 3:** Genotypic and allelic analyses of prostate cancer susceptibility

Genotype		Control vs. SPCa			Control vs. LPCa	
	OR*	95% CI	*p *value	OR*	95% CI	*p *value
rs1447295						
CC	1.0	reference		1.0	reference	
CA	1.54	1.08 – 2.21	0.02	1.22	0.75 – 1.98	0.42
AA	1.29	0.62 – 2.73	0.50	1.45	0.53 – 3.59	0.45
CA + AA	1.50	1.07 – 2.11	0.02	1.25	0.79 – 1.97	0.33
rs6983267						
TT	1.0	reference		1.0	reference	
TG	0.95	0.67 – 1.35	0.78	1.17	0.74 – 1.86	0.50
GG	2.21	1.24 – 4.03	7.0 × 10^-3^	1.40	0.62 – 3.03	0.40
TG + GG	1.12	0.80 – 1.57	0.50	1.21	0.78 – 1.88	0.41

Allele						
	OR*	95% CI	*p *value	OR*	95% CI	*p *value

rs1447295						
C	1.0	reference		1.0	reference	
A	1.34	1.01 – 1.79	0.04	1.23	0.84 – 1.79	0.29
rs6983267						
T	1.0	reference		1.0	reference	
G	1.27	0.99 – 1.62	0.06	1.17	0.84 – 1.62	0.35

OR*, Age-adjusted odds ratio.

After stratification according to the Gleason score, T-stage, and serum prostate specific antigen (PSA) levels at the time of diagnosis, the frequencies of the CA and CA+AA genotypes of rs1447295 were observed significantly higher in SPCa patients with a Gleason score ≤7, a T-stage of T3 or T4, and a serum PSA level ≥20 ng/mL; meanwhile, the frequency of the GG genotype of rs6983267 was observed significantly higher in SPCa patients with a Gleason score ≥8, a T-stage of T1 or T2, and a serum PSA level ≥20 ng/mL (Table 4). We also investigated a combined model examining the joint effect of both rs1447295 and rs6983267 on the prostate cancer risk. Individuals with SPCa who carried both the CA (rs1447295) and the GG (rs6983267) genotypes had a significantly higher risk of prostate cancer (*p *= 0.03; age-adjusted OR, 2.74; 95% CI, 1.13 – 7.17) (Table 5).

**Table 4:** Prostate cancer susceptibility stratified by Gleason score, T-stage, and serum PSA levels at diagnosis (control vs. SPCa)

Genotype	Gleason score ≤ 7 (N = 241)			Gleason score ≥ 8 (N = 150)		
	OR*	95% CI	*p *value	OR*	95% CI	*p *value
rs1447295						
CC	1.0	reference		1.0	reference	
CA	1.62	1.08 – 2.42	0.02	1.48	0.94 – 2.34	0.09
AA	1.09	0.46 – 2.56	0.84	1.63	0.66 – 3.92	0.29
CA + AA	1.53	1.04 – 2.25	0.03	1.50	0.97 – 2.32	0.07
rs6983267						
TT	1.0	reference		1.0	reference	
TG	0.93	0.62 – 1.39	0.73	0.90	0.57 – 1.42	0.66
GG	1.84	0.96 – 3.55	0.07	2.41	1.20 – 4.86	0.01
TG + GG	1.05	0.72 – 1.55	0.79	1.10	0.72 – 1.69	0.67

Genotype		T1 or T2 (N = 167)			T3 or T4 (N = 224)	
	OR*	95% CI	*p *value	OR*	95% CI	*p *value

rs1447295						
CC	1.0	reference		1.0	reference	
CA	1.39	0.88 – 2.17	0.15	1.70	1.14 – 2.56	0.01
AA	1.34	0.53 – 3.29	0.53	1.28	0.55 – 2.95	0.57
CA + AA	1.38	0.90 – 2.11	0.14	1.64	1.11 – 2.41	0.01
rs6983267						
TT	1.0	reference		1.0	reference	
TG	0.96	0.61 – 1.51	0.87	0.91	0.61 – 1.36	0.65
GG	2.68	1.37 – 5.30	4.0 × 10^-3^	1.69	0.87 – 3.31	0.12
TG + GG	1.19	0.78 – 1.83	0.42	1.02	0.69 – 1.49	0.93

Genotype		PSA < 20 ng/mL (N = 208)			PSA ≥ 20 ng/mL (N = 183)	
	OR*	95% CI	*p *value	OR*	95% CI	*p *value

rs1447295						
CC	1.0	reference		1.0	reference	
CA	1.46	0.95 – 2.23	0.08	1.71	1.12 – 2.60	0.01
AA	0.97	0.37 – 2.44	0.95	1.69	0.74 – 3.83	0.21
CA + AA	1.38	0.92 – 2.08	0.12	1.70	1.14 – 2.54	9.0 × 10^-3^
rs6983267						
TT	1.0	reference		1.0	reference	
TG	0.96	0.63 – 1.47	0.86	0.92	0.60 – 1.40	0.69
GG	1.46	0.72 – 2.96	0.30	2.74	1.45 – 5.24	2.0 × 10^-3^
TG + GG	1.03	0.69 – 1.55	0.88	1.16	0.78 – 1.73	0.47

Genotype		Nonaggressive (N = 99)			Aggressive^a^ (N = 292)	
	OR*	95% CI	*p *value	OR*	95% CI	*p *value

rs1447295						
CC	1.0	reference		1.0	reference	
CA	1.69	0.98 – 2.91	0.06	1.54	1.06 – 2.25	0.02
AA	1.39	0.44 – 3.99	0.56	1.28	0.59 – 2.81	0.53
CA + AA	1.64	0.97 – 2.75	0.06	1.50	1.05 – 2.15	0.03
rs6983267						
TT	1.0	reference		1.0	reference	
TG	0.99	0.58 – 1.71	0.97	0.93	0.64 – 1.36	0.72
GG	1.45	0.58 – 3.46	0.42	2.32	1.29 – 4.26	0.01
TG + GG	1.05	0.63 – 1.78	0.85	1.12	0.79 – 1.60	0.52

OR*, Age-adjusted odds ratio.

^a ^Aggressive, Gleason score ≥ 8, and/or T-stage T3 or T4, and/or PSA ≥ 20 ng/mL.

**Table 5:** Combined analysis with two polymorphisms

		rs6983267		
		TT	TG	GG
	
rs1447295	OR*	1.0	0.78	1.69
CC	95% CI	reference	0.50 – 1.23	0.78 – 3.75
	OR*	1.07	1.33	2.74
CA	95% CI	0.58 – 1.94	0.77 – 2.29	1.13 – 7.17
	OR*	0.54	1.34	－
AA	95% CI	0.15 – 1.81	0.42 – 4.48	－

OR*, Age-adjusted odds ratio.

—, not applicable.

## Discussion

The present case-controlled study investigated the relation between variants on human chromosome 8q24 and the risk of sporadic and latent prostate cancer in native Japanese men. We confirmed that the rs1447295 and rs6983267 polymorphisms were significantly associated with the risk of sporadic prostate cancer in native Japanese men. Table 6 shows the results of allele-wise analyses for prostate cancer susceptibility in multi-ethnic studies. The frequencies of the risk alleles and the magnitudes of the effects found in our study were very similar to those previously reported for Japanese Americans [3,8]. Neither rs1447295 nor rs6983267 was associated with the risk of prostate cancer in African Americans; however, positive associations with other genetic markers on chromosome 8, such as DG8S737 [2,8], rs16901979 [7,11], rs13254738, rs6983561, broad11934905, rs7000448 [8], and rs7008482 [11], were also reported in these subjects. Whether these markers might also be valid for native Japanese men should be confirmed in future studies. In a genome-wide association study in subjects of European ancestry [9], the ORs of the CA and AA genotypes of rs1447295 were 1.43 and 2.23, respectively. In our results, patients with the CA genotype of rs1447295 were more likely to have prostate cancer (*p *= 0.02; age-adjusted OR, 1.54; 95% CI, 1.08–2.21); however, the AA genotype was not significantly associated with prostate cancer in the present study (*p *= 0.50; age-adjusted OR, 1.50; 95% CI, 0.62–2.73). In addition, the CA+AA genotypes of rs1447295 were observed more frequently in the SPCa group (*p *= 0.02; age-adjusted OR, 1.50; 95% CI, 1.07–2.11), similar to the results of a previous study conducted in Australian men [4]. As for the rs6983267 polymorphism, we could not find any significant association between the G allele and SPCa (*p *= 0.06; age-adjusted OR, 1.27, 95% CI: 0.99–1.62), although Haiman et al. [8] reported a significant association between these two factors (OR, 1.22; 95%CI, 1.05–1.42) in a study sample of 1,450 Japanese-Americans (722 patients with SPCa and 728 controls). The disparity between the results of their report and ours might have been caused by the different sample sizes.

**Table 6:** Allele-wise analyses for prostate cancer risk in the multiethnic studies

			rs1447295				
			A allele frequency				
Author (Year)	*N *cases/*N *controls	Study population	Cases	Controls	OR	95% CI	*p *value

Amundadottir, et al. (2006)	1291/997	Iceland	0.17	0.11	1.72	—	1.7 × 10^-9^
	1435/779	Sweden	0.16	0.13	1.29	—	4.5 × 10^-3^
	458/247	European Americans	0.13	0.08	1.66	—	6.7 × 10^-3^
	246/352	African Americans	0.34	0.31	1.15	—	0.29
Freedman, et al. (2006)	70/68	Native Hawaiians	0.37	0.16	3.02	1.66 – 5.50	1.5 × 10^-4^
	449/465	Japanese Americans	0.24	0.17	1.48	1.18 – 1.86	3.4 × 10^-4^
	640/567	Latino Americans	0.14	0.10	1.48	1.14 – 1.91	1.4 × 10^-3^
	455/447	European Americans	0.13	0.10	1.35	1.01 – 1.80	0.02
	989/804	African Americans	—	—	1.05	0.95 – 1.16	0.15
Wang, et al. (2007)	491/545	Caucasians	0.12	0.10	1.16	0.85 – 1.58	0.25
	435/545	Caucasians*	0.17	0.10	1.93	1.37 – 2.72	4.0 × 10^-3^
Gudmundsson, et al. (2007)	1453/3064	Iceland	0.17	0.10	1.71	1.49 – 1.95	1.6 × 10^-14^
	385/892	Spain	0.10	0.07	1.44	1.07 – 1.94	1.7 × 10^-2^
	367/1302	The Netherlands	0.14	0.11	1.39	1.09 – 1.78	9.0 × 10^-3^
	373/372	African Americans	0.32	0.31	1.01	0.81 – 1.25	0.96
Haiman, et al. (2007)	1614/837	African Americans	—	—	—	—	—
	722/728	Japanese Americans	—	—	—	—	—
	111/112	Native Hawaiians	—	—	—	—	—
	637/633	Latino Americans	—	—	—	—	—
	1182/942	European Americans	—	—	—	—	—
Robbins, et al. (2007)	490/567	African Americans	0.34	0.31	1.40	0.70 – 1.30	0.13
Present study (2009)	391/323	Native Japanese	0.25	0.19	1.34	1.01 – 1.79	0.04

			rs6983267				
			G allele frequency				

Author (Year)	*N *cases/*N *controls	Study population	Cases	Controls	OR	95% CI	*p *value

Amundadottir, et al. (2006)	1291/997	Iceland	—	—	—	—	—
	1435/779	Sweden	—	—	—	—	—
	458/247	European Americans	—	—	—	—	—
	246/352	African Americans	—	—	—	—	—
Freedman, et al. (2006)	70/68	Native Hawaiians	—	—	—	—	—
	449/465	Japanese Americans	—	—	—	—	—
	640/567	Latino Americans	—	—	—	—	—
	455/447	European Americans	—	—	—	—	—
	989/804	African Americans	—	—	—	—	—
Wang, et al. (2007)	491/545	Caucasians	—	—	—	—	—
	435/545	Caucasians*	—	—	—	—	—
Gudmundsson, et al. (2007)	1453/3064	Iceland	—	—	—	—	—
	385/892	Spain	—	—	—	—	—
	367/1302	The Netherlands	—	—	—	—	—
	373/372	African Americans	—	—	—	—	—
Haiman, et al. (2007)	1614/837	African Americans	0.90^†^	0.84	1.43	1.17 – 1.75	—
	722/728	Japanese Americans	0.37^†^	0.32	1.22	1.05 – 1.42	—
	111/112	Native Hawaiians	0.36^†^	0.28	1.29	0.88 – 1.89	—
	637/633	Latino Americans	0.63^†^	0.62	1.05	0.89 – 1.24	—
	1182/942	European Americans	0.54^†^	0.51	1.13	0.99 – 1.28	—
Robbins, et al. (2007)	490/567	African Americans	0.08	0.11	1.40	0.90 – 2.40	0.17
Present study (2009)	391/323	Native Japanese	0.38	0.31	1.27	0.99 – 1.62	0.06

—, not applicable.

*, Familial prostate cancer cases.

†: We calculated the G allele frequencies of rs6983267. Genotype data were obtained from the supplementary Table 3 (reference No. 8).

In 2008, Terada et al. [16] initially studied 507 patients with prostate cancer and 511 controls to determine the association between these common variants on chromosome 8q24 and the risk of prostate cancer in a native Japanese population. For their Japanese subjects, the A allele of rs1447295 was significantly associated with the risk of prostate cancer, while the G allele of rs6983267 was associated with the risk of prostatic enlargement rather than prostate cancer. Their findings for rs6983267 differed from the results reported by Haiman et al. regarding the risk of prostate cancer in Japanese Americans [8]. Terada et al. adopted healthy male volunteers or patients of benign disease as control, while our controls were pathologically confirmed to be the cases which have not suffered from any malignancy by autopsy. We could not read from their report whether they had surveyed of controls by a PSA test or physical examination to exclude prostate cancer subjects. We worried that they had a potential chance including prostate cancer subjects among controls and patients with benign prostatic hyperplasia. We think careful setting of controls is a critical issue that interferes with the results. During the course of our experiment, we also observed a relation between the aggressiveness of SPCa and these two SNPs. Thus, we investigated possible interactions between these two independent SNPs. Our results revealed a significant OR only in patients carrying both the CA genotype of rs1447295 and the GG genotype of rs6983267. While this result differed from that reported by Yeager et al. [9] in a European population, the magnitude of the effect in our study was higher (age-adjusted OR, 2.74; 95% CI, 1.13–7.17). The accumulation of samples in collaboration with various international centers would be indispensable for the further study of such interactions.

The functional features of these polymorphisms are still unclear. The closest characterized gene is the proto-oncogene *c-MYC*, located approximately 264 kb from rs1447295 [2,6]. Sato et al. [17] demonstrated that patients with additional increases in the *c-MYC *copy number relative to the chromosome 8 centromere showed a more rapid disease progression and earlier death from cancer. Thus, clarifying the relation between *c-MYC *amplification and the risk alleles among the polymorphic markers located in 8q24 is a crucial task.

To the best of our knowledge, no study to date has examined common variants on human chromosome 8 among subjects with LPCa. Our results suggested that the rs1447295 and rs6983267 polymorphisms were not associated with the risk of LPCa in native Japanese men. The frequencies of each genotype in these subjects and the controls were quite similar. We believe that such genetic research in subjects with LPCa may be useful for clarifying the genetic characteristics of clinically insignificant prostate cancer.

In Japan, the age-adjusted incidence rate of prostate cancer was determined to be 26.2 per 100,000 in 2001 [18], while that in Japanese Americans living in California was determined to be 103.7 per 100,000 (2000 to 2002) [1]. Denis et al. [19] suggested that low intakes of vitamin E, selenium, lignans and isoflavonoids affected the tumorigenesis risk of prostate cancer. Marks et al. [20] reported that Japanese Americans had more body fat, higher serum triglyceride levels, lower estradiol levels, and much lower soy-metabolite levels than native Japanese. Additionally, Bettuzzi et al. [21] and Kurahashi et al. [22] emphasized the protective effects of green tea constituents against the risk of prostate cancer. These findings suggest that we need to consider genetic and environmental factors, as well as nutrition, in our attempts to prevent prostate cancer.

This study had several limitations. First, our sample size was relatively small, and this might have influenced the robustness of the results. Further study using larger samples for both the case and control groups is necessary. Secondary, several pathologists at the affiliated hospitals had participated in the diagnosis of prostate cancer. Therefore, we cannot deny the inter-observer variability existed. Moreover, the data of Gleason score was underestimated, because two thirds of the pathological data from SPCa patients were obtained by biopsy.

Little is known about why the prevalence of prostate cancer is much lower in Asian populations than in other ethnicities. Researchers from Asia need to collaborate in addressing this issue.

## Conclusion

The present study demonstrates that polymorphisms on 8q24 are associated with the occurrence of prostate cancer in the native Japanese population. Our results confirm that these polymorphisms are closely related to the risk of SPCa; however, we did not observe an association between these polymorphisms and the risk of LPCa. One limitation of our study was that the number of our samples was relatively small; consequently, studies with larger populations and family-based analyses are needed in the future.

## Methods

### Study design

A total of 391 Japanese patients with SPCa who were treated at the Department of Urology at the University of Tokyo Hospital or at our affiliated hospitals located in the Kanto area of Japan between January 1999 and August 2007 were enrolled. One hundred twenty-six cases underwent a radical prostatectomy and the remaining cases were treated with androgen-deprivation therapy. Adenocarcinoma of the prostate was pathologically confirmed in all the cases, and the Gleason score was also evaluated by pathologists working at each hospital. The clinical T stage of the patients with SPCa was evaluated based on a digital rectal examination, transrectal ultrasonography, pelvic computed tomography, and pathological findings according to the 2002 TNM staging system for cancer [23]. The serum PSA levels at diagnosis were also measured. Patients with a family history of prostate cancer were carefully excluded from this study. The mean age of the SPCa patients was 70.7 ± 8.0 years (median, 71 years; range, 48 to 89 years). Genomic DNA samples were extracted from peripheral blood specimens of these patients. The study was conducted with the approval of the Ethics Committee of the University of Tokyo and the internal review board of each of the affiliated hospitals. Written informed consent was obtained from each patient prior to their enrollment in the study. We also examined 323 residence-matched Japanese men as a control group. The mean age of the patients in the control group was 79.2 ± 9.2 years (median, 79 years; range, 49 to 100 years). All the patients in the control group had died at the Tokyo Metropolitan Geriatric Hospital and were consecutively autopsied. All the control patients were pathologically confirmed to not have suffered from any malignancy [24]. Moreover, we examined 112 Japanese men who were diagnosed as having LPCa at the time of autopsy. The mean age of the LPCa patients was 81.9 ± 7.6 years (median, 81 years; range, 66 to 98 years). These patients had been registered in the database of Japanese single nucleotide polymorphisms for geriatric research (JG-SNP) [25]. Genomic DNA samples from the controls and LPCa cases were extracted from frozen kidney tissues. The T stage of the patients with LPCa was evaluated based on pathological findings according to the 2002 TNM staging system for cancer [23]. Written informed consent was obtained from the patients' family members under the Act of Postmortem Examination. This study was also reviewed and approved by the Ethics Committee of the Tokyo Metropolitan Geriatric Hospital. Moreover, this study was carried out in compliance with the Helsinki Declaration.

### Genotyping assay

Genotyping of the rs1447295 and rs6983267 polymorphisms was conducted using a TaqMan assay with an ABI PRISM 7000 or 7300 sequence detection system (Applied Biosystems, Foster City, CA, USA) according to the manufacturer's instructions. We adjusted the concentration of the DNA solution to 100 ng/mL before using it. The sequences of the specific primers and TaqMan probes and the conditions for quantitative real-time polymerase chain reaction were obtained from the website of the National Cancer Institute .

### Statistical analysis

All the statistical analyses were conducted using JMP software, version 7.0 (SAS, Cary, NC, USA). The chi-square test was used to examine Hardy-Weinberg equilibrium (HWE) and to compare the distribution of the genotypes and alleles among the control, SPCa and LPCa patients. To estimate the odds ratios (ORs) and 95% confidence intervals (CIs), logistic regression analyses were performed using age as a covariate to statistically adjust for its potential confounding effects. Additionally, we estimated ORs and 95%CIs after stratification according to the Gleason score, T-stage, and serum PSA levels at diagnosis, as just as Terada et al. reported [16]. P values less than 0.05 were considered significant.

## Abbreviations

LPCa: latent prostate cancer diagnosed at the time of autopsy; SPCa: clinically diagnosed sporadic prostate cancer; HWE: Hardy-Weinberg equilibrium; PSA: prostate specific antigen; OR: odds ratio; CI: confidence interval.

## Authors' contributions

ML carried out the genotyping assay, data analysis, and drafted the manuscript. TK1 carried out the genotyping assay. YE analyzed and interpreted the data. TA, MS2, and TH collected the control and latent prostate cancer subjects and interpreted the data. TK2 and MS1 conceived the study. MS1 was also involved in the statistical analysis and drafting of the manuscript. HN was involved in the manuscript's revision. TK2 and YH approved the manuscript for publication. All authors read and approved the final manuscript.
